# A randomized controlled trial protocol for persistent physical symptoms associated with indoor environment or chronic fatigue: Effectiveness of video-based functional case conceptualization and web-program for improving quality of life

**DOI:** 10.3389/fpsyg.2022.923532

**Published:** 2023-01-06

**Authors:** Sanna Selinheimo, Katariina Keinonen, Aki Vuokko, Sanna Liesto, Markku Sainio, Raimo Lappalainen, Tiina Paunio

**Affiliations:** ^1^Finnish Institute of Occupational Health, Helsinki, Finland; ^2^Outpatient Clinic for Functional Disorders, HUS Helsinki University Hospital, Helsinki, Finland; ^3^Department of Psychology, University of Jyväskylä, Jyväskylä, Finland; ^4^Department of Psychiatry and SleepWell Research Program, Faculty of Medicine, Helsinki University Central Hospital, University of Helsinki, Helsinki, Finland

**Keywords:** persistent physical symptoms, intervention, eHealth, personalization, chronic fatigue syndrome, indoor air, environmental intolerance

## Abstract

**Introduction:**

Persistent physical symptoms (PPS) refer to symptoms that cannot be fully explained by structural bodily pathology or by environmental factors. Their impact on daily functioning varies from mild to severe disability. So far, evidence-based treatments for PPS have resulted in only small to moderate effects. Treatment protocols with a stronger orientation toward personalized approaches are needed to improve the efficacy and applicability of treatment. In this study, we aim to assess the effect of an online individual case conceptualization with web-based program for PPS. This study is conducted among two focus groups: patients with indoor air-related symptoms and patients with chronic fatigue syndrome.

**Methods and analyses:**

Using a randomized controlled design (RCT) with two parallel groups in a 1:1 ratio, we will compare individual video-based case conceptualization with a web-based program based on Acceptance and Commitment Therapy (ACT), combined with treatment as usual, with treatment as usual only. The web-based program consists of ten modules, each lasting 1 week and including training. The planned sample size is 124 eligible patients without attrition. The primary outcome will be the health-related quality of life as measured by the 15D questionnaire. The secondary outcome measures will include questionnaires on psychiatric and physical symptoms, illness perceptions, psychological flexibility, and work ability. We will also use national registers to obtain information on the use of healthcare and social benefits to complete patient-reported outcomes. Data collection began in August 2020 and will continue until 2023.

**Discussion:**

This trial will provide information on the effects and usefulness of an online administrated individual case conceptualization and an ACT-based web-program on PPS.

**Ethics and dissemination:**

The Ethics Committee of the Hospital District of Helsinki and Uusimaa, Finland, has granted approval for the study. The results will be published in peer-reviewed journals.

**Clinical Trial Registration:**

Clinicaltrials.gov, identifier NCT04532827 preresults.

## 1. Introduction

Frequent somatic symptoms are common among the general population, with over 90% reporting symptoms at some level (Hiller et al., 2006; Eliasen et al., 2016). It is not uncommon that symptoms to become persistent and reduce work ability and daily functioning (Hiller et al., 2006; Aamland et al., 2012; Rask et al., 2015). Persistent somatic symptoms have been associated with increased somatic or psychiatric comorbidity (Henningsen et al., 2003; Haug et al., 2004; Petersen et al., 2018; Selinheimo et al., 2019), which increases the burden on healthcare and the risk of sick leaves or long-term work disability (Aamland et al., 2012; Loengaard et al., 2015; Rask et al., 2015) also independently of these comorbidities (Barsky et al., 2005). Self-perceived health is a strong predictor of an increased risk of work disability (Airaksinen et al., 2017), and persistent physical symptoms (PPS) challenge healthcare systems. The prevalence rates of PPS without a clear medical explanation in primary healthcare patients have been shown to range from 1.6% up to 49% (Kirmayer et al., 2004; Hilderink et al., 2013; Haller et al., 2015; Loengaard et al., 2015) and tertiary healthcare visits show similar estimations (Nimnuan et al., 2001).

PPS associated with chronic fatigue syndrome or environmental factors has been linked to impaired quality of life and significant disability both at work and in people’s personal lives (Cairns and Hotopf, 2005; Leone et al., 2006; Karvala et al., 2013; Vuokko et al., 2015; Selinheimo et al., 2019). A part of the general population’s PPS is associated with environmental factors such as indoor air at pollutant levels far below those toxicologically established as causing harmful effects on daily life, or PPS continues despite reparations to indoor environments (Ross et al., 2004; Lacour et al., 2005; Das-Munshi et al., 2007; Norbäck, 2009; Van den Bergh et al., 2017a). The key feature of chronic fatigue syndrome is a substantial reduction in the ability to engage in pre-illness levels of activity accompanied by excessive fatigue after physical, mental, or emotional exertion, and that fatigue is not substantially alleviated by rest (Jason et al., 2010). These conditions share multi-organ symptom profiles and the characteristics of a discrepancy between objectively assessed and subjective health and based on current understanding, PPS with different bodily symptom manifestations share similar processes that trigger and maintain the outcomes (Burton et al., 2020). However, predisposing and precipitating factors are only partially known and may vary between individuals, which challenges the rehabilitation targeting of these conditions (Ross et al., 2004; Norbäck, 2009; Van den Bergh et al., 2017a,b; Henningsen et al., 2018).

Treating PPS has been challenging because of both the ambiguous definition of the condition and the divergent views of patients and clinicians on the mechanisms of the condition. These discrepancies are reflected in the acceptance of treatment modalities and the aims of treatments, resulting in frustration and feelings of being misunderstood among both patients and professionals (Olde Hartman et al., 2009; Peters et al., 2009; van Ravenzwaaij et al., 2010; Adamowicz et al., 2014; Pemberton and Cox, 2014; Tuuminen et al., 2016; Vuokko et al., 2016). Psychosocial, patient-involving methods, such as cognitive-behavioral psychotherapy (CBT), which support individuals’ abilities to manage their symptoms and health behaviors, have shown promising effects in supporting the functional ability and quality of life of individuals with PPS (Henningsen et al., 2018). However, the effect sizes have remained rather moderate and the information on factors explaining the variability of individuals´ responses to treatment is scarce (Kleinstauber et al., 2011; van Dessel et al., 2014). It has also been suggested that the rationale behind the psychosocial treatments remains unclear for the patients and thus results in unwanted effects such as withdrawal from the treatment (Geraghty and Scott, 2020). To respond to these challenges some studies have suggested that tailoring treatments according to personalized profiles might improve the effectiveness (Rosmalen et al., 2020; Senger et al., 2021a,b). These profiles include both state characteristics such as the severity of the symptoms and comorbidities but also lifestyle factors and cognitions and emotions that contribute to the response to treatment. If aiming to improve personalization, these characteristics should be evaluated when referring to the treatment. The acceptability of these treatments among symptomatic individuals is also low—presumably because of the stigma related to psychological treatments and the vague definition of the condition (Åsbring and Närvänen, 2002; Looper and Kirmayer, 2004; Dickson et al., 2007; Finell et al., 2018). As adherence is suggested to improve the treatment effect on health even more than improvements in specific treatments methods (Brown and Bussell, 2011; Donkin et al., 2011), more information on the tools for increasing personalized approaches and rationale for treatments and acceptability for patients is urgently needed to improve clinical practices and to enhance patients’ functioning.

Traditional psychosocial treatments may favor individuals who are more willing and able to consult traditional healthcare services (van Gils et al., 2016), and thus Internet-delivered treatments may be provided to individuals who regard traditional treatments as stigmatizing. Moreover, Internet-based treatments with minimal therapist contact might also offer an alternative to psychosocial treatments for individuals in cases of recurrent poor experiences of healthcare (Henderson et al., 2013; van Gils et al., 2016) that are common in PPS associated with chronic fatigue or environmental factors.

Acceptance and commitment therapy (ACT) is a form of CBT that aims to improve psychological flexibility, or the ability to commit to acting in line with one’s values to enhance the quality of life while using acceptance-based strategies to react to inner experiences (Hayes et al., 2009). ACT is based on the Relational Frame Theory (RFT), a contextual behavioral approach to cognitions that proposes that changing the function of private events is more viable for changing the content of those events (Törneke et al., 2008). Several meta-analyses have also supported the effectiveness of ACT in various populations, including mood and anxiety disorders, addictive behaviors, and several health-related problems such as chronic pain (e.g., Powers et al., 2009; Ruiz, 2010) in Internet-delivered settings in cases of presumable barriers to traditional treatments (Brown et al., 2016; Kelson et al., 2019). In addition, some data show that ACT can improve quality of life and decrease fatigue among patients with chronic fatigue in inpatient rehabilitation settings (Jacobsen et al., 2017) and further, that acceptance has a direct or an indirect effect on wellbeing and functioning among individuals with chronic fatigue syndrome (Van Damme et al., 2006; Roche et al., 2017). To the best of the authors’ knowledge, previous research on ACT among patients with PPS associated with indoor air is not available, and the information on effective treatments for this population of patients is scarce.

### 1.1. Objectives

The aim of this randomized controlled trial (RCT) is to investigate the effectiveness of individual case formulation by using a functional case conceptualization method and shared goal-setting for the treatment, combined with an ACT web-based program, in comparison to treatment as usual (TAU) for disabling PPS among individuals with PPS related to i) indoor environments or ii) chronic fatigue syndrome. Its´ secondary aims are to assess the interaction of individual reaction patterns and processes based on the theoretical model of psychological flexibility with the intervention’s effectiveness and to investigate whether the intervention decreases participants’ overall symptom burden and improves daily functioning. We will also explore the participants’ adherence and response to the treatment to increase our understanding of the acceptability of the interventions for PPS.

## 2. Methods and analysis

### 2.1. Study design

This study is a randomized controlled superiority trial of two parallel groups. It is carried out in Finland by the Finnish Institute of Occupational Health (FIOH) in collaboration with the University of Jyväskylä, the University of Helsinki, and the Hospital District of Helsinki and Uusimaa between 2020 and 2023. Recruitment information of the participants began at the end of August 2020 and the first clinical interview for inclusion was conducted at the beginning of September 2020.

### 2.2. Participant recruitment channels and enrolment in the study

Participants are recruited through several sources. Firstly, occupational health service (OHS) units were contacted *via* the national network of OHS providers and *via* the chief medical doctors of primary healthcare and OHS units. The study is further advertised in healthcare specialists’ journals, on national training days for medical doctors, and by sending emails to members of unions, focusing on primary and occupational health doctors. Further, we will contact hospital rehabilitation units and clinics specialized in indoor environment-associated symptoms or chronic fatigue syndrome in University hospitals across Finland. The informed medical doctors will assess the eligibility of participant candidates and recommend the study to eligible individuals. The reduction of healthcare utilization due to the COVID-19 pandemic influenced participant recruitment *via* healthcare units. During the first 5 months, eight eligible participants were recruited. Therefore, to not overextend the recruitment period, two amendments to the recruitment channels were accomplished. First, the recruitment channels from healthcare were extended to Finnish Student Health Service units in February 2021, after agreement with the study steering group (November 2020), and approval from Ethics Committee (January 2021), Secondly, participants experiencing chronic fatigue (see inclusion criteria: Table 1) were further recruited among a cohort of individuals with prolonging post-COVID-19 symptoms. Helsinki University Hospital conducted a questionnaire survey in January 2022 for all non-hospitalized individuals living on its´ medical treatment circuit who had positive COVID-19 test results from the laboratory during March 2021. A cover letter with the link to an online questionnaire was sent to a survey cohort. After filling in the online questionnaire, it automatically informed those individuals who reported persistent fatigue (≥6 months) that influenced their ability to work or study of this RCT. The study steering group agreed on the amendment in May 2021 and it was approved by the Ethics Committee in July 2021.

**Table 1:** Inclusion and exclusion criteria of the study.

Criteria	Description
**Inclusion**	
Age and gender	Age 18 to 65 years, all genders
Language	Fluent Finnish
Duration of symptoms	Onset of symptoms with disability of 3 years maximum before the study
Symptomatology (A) Indoor air-related symptoms (IPCS/WHO, 1996; Lacour et al., 2005)	(A) Indoor air-related symptoms
	(a) Self-reported symptoms attributed to indoor (non-industrial) environments including: (i) symptoms in at least two different organ systems, e.g., respiratory, digestive, or nervous system
	(b) Symptoms recurring (i) in more than one indoor environment or (ii) despite environmental improvements (e.g., work arrangements and/or workplace reparations)
(B) Chronic fatigue syndrome (ME/CFS; Jason et al., 2010)	(B) Chronic fatigue
	(a) Post-exertional malaise and/ or post-exertional fatigue
	(b) Unrefreshing sleep or disturbance of sleep quantity or rhythm disturbance
	(c) Pain, often widespread
	(d) Two or more neurological or cognitive symptoms
	(e) At least two symptoms from the following categories (i) Autonomic manifestations, (ii) Neuroendocrine manifestations or (iii) Immune manifestations
Duration and severity of condition	Minimum of 6 months; Symptoms are not lifelong and result in substantial functional restrictions in daily life
Occupation	All occupations
**Exclusion**	
Work situation	Long sick leave (≥3 months) without return-to-work plan, not actively participating in the study or work life (retired or unemployed)
Medical reasons	(a) Some serious and/or acute medical disease or illness that explains the symptoms (i) Somatic disease that explains the symptoms (e.g., uncontrolled asthma, hypothyroidism, and sleep apnea)
	(ii) Psychiatric disorder (bipolar disorder, psychotic disorders, alcohol and/or drug dependency or abuse, eating disorders, and/or severe mood disorders)
	(b) Developmental disorders
Psychotherapy	Psychotherapy (current)
Other	Patient refusal

Participants are further recruited through local newspapers, employee support organizations (sectors of teaching, social welfare, and healthcare), and patient organizations (asthma and allergy associations) using both print and social media.

Before enrolment in the study, all participant candidates independently from the recruitment channel will inform their interest in the study *via* e-form and they will receive oral and written information on the study from the study researchers (SS or KK). After receiving information, participant candidates will enroll for the study by filling in an electronic informed consent.

### 2.3. Eligibility criteria

**Figure 1:**
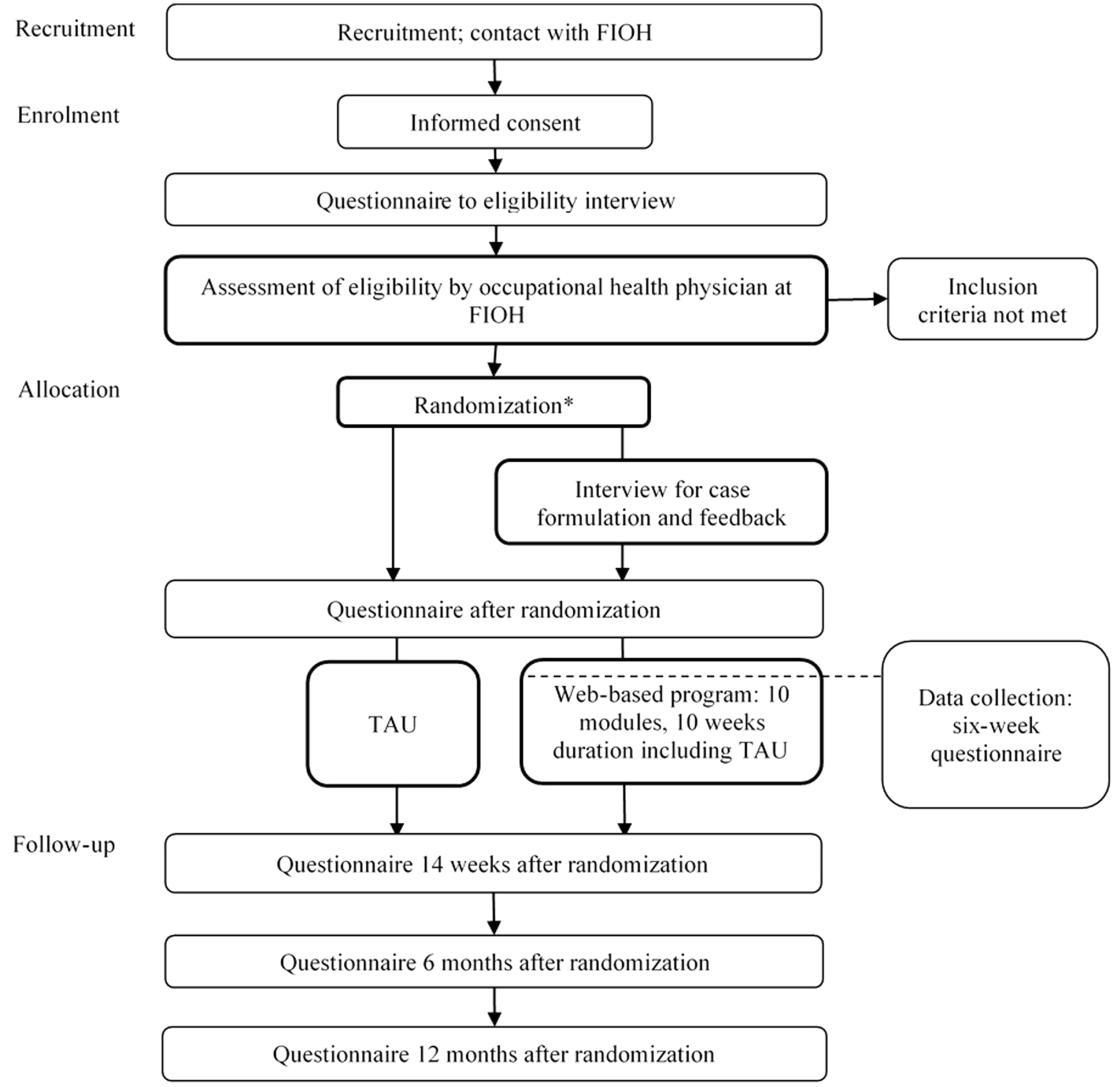
Participant flowchart. FIOH, Finnish Institute of Occupational Health; TAU, treatment as usual. *After randomization all participants receive an educational leaflet.

Participants will need to be aged 18–65 years and provide informed consent. As online intervention can be considered as low-intensity first-line treatment, participants will need to be occupationally active or study actively (university or applied university) indicating a low level of disability. They must have PPS associated with indoor environments or chronic fatigue syndrome, which was defined according to the criteria of myalgic encephalomyelitis/chronic fatigue syndrome (Jason et al., 2010). Participants will enroll at the Finnish Institute of Occupational Health, where they will undergo structured, clinical video-based interviews about the inclusion and exclusion criteria. Table 1 shows detailed symptom definitions (see IPCS/WHO, 1996; Lacour et al., 2005; Jason et al., 2010). Figure 1 outlines the participant flow, data collection, and intervention program timeline.

### 2.4. Patient and public involvement

Neither the patients nor the public were involved in planning or developing the study design, research questions, selection of the outcome measures, or study conduct. Volunteer patient representatives, i.e., individuals with expert experience, participated in evaluating the acceptability of the pilot version of the web-based program intervention by reviewing the manuscript and providing feedback. The pilot version was further developed after 11 months of usage (see further information in the section *eHealth intervention*).

### 2.5. Clinical interview

Medical doctors registrar to occupational medicine will conduct manualized and structured 45–60-min video-based clinical interviews to ensure the participants’ eligibility. These individual interviews, modified versions of the semi-structured Research Interview for Functional somatic Disorders (RIFD) interview (Petersen et al., 2019), will be used to identify the multiplicity and course of the following symptom clusters or disorders: cardiopulmonary, gastrointestinal, musculoskeletal, neurological, general, and other symptoms; fatigue; environmental intolerance; health anxiety; depression; anxiety; and other mental disorder. During the interview, the interviewer will assess whether or not a symptom or symptom pattern is present, the severity of the symptoms and impairment related to the symptoms and the time when symptoms extended to disabling level, and possible comorbid medical conditions that may account for the individual’s symptomatology and disability. Data from the questionnaire before the interview will be used as a base for the interviews (Table 4). Participants requiring medical care or further medical examinations (i.e., a suspect of an untreated medical condition that might explain the symptoms arising during the interview) verified by the interview will be referred to a healthcare professional. If they meet the inclusion criteria (Table 1), the participants will be randomized into TAU or eHealth intervention groups, including TAU enhanced with individual case formulation, and a 10-week web-based program for PPS.

### 2.6. Intervention: Video-based individual case conceptualization and goal-setting using the web-based program

The intervention will start with two video meetings with a psychologist to build and present an individual functional case conceptualization (Tuomisto et al., 1998; Haynes and O'brien, 2000) and reach a shared decision on individual goals and treatment targets for the web-based program. Psychologists (KK, SS) will deliver these manualized 45–60-min individual sessions. The first session will include an interview (Strosahl et al., 2012) considering the participant’s psychosocial situation to establish the individual’s symptomatology and current life situation. The psychologist will build a case conceptualization based on the interview. The case conceptualization will be presented and discussed during the second session and necessary modifications can be made to ensure acceptability for the participant. Individual goals will be set based on the approved case formulation and understanding of functional relationships among factors contributing to the individual’s wellbeing.

The web-based program will be offered after the meeting with the psychologist and will consist of 10 manualized modules at 1-week intervals. Participants will be instructed to complete each module and to continue to integrate the content into their daily lives during the following weeks. The modules will include psychoeducation and experiential exercises and training aimed at improving wellbeing and psychological flexibility following the contextual behavioral approach to wellbeing (Table 2). All the modules will include experiential exercises. The participants will receive weekly written feedback on each module from psychologists, nurses, and a social worker who will act as therapists in the study. These therapists are employed by the Hospital District of Helsinki and Uusimaa and are trained in providing web-based interventions to various populations. In addition to written feedback, the therapists will call all the participants and give them instructions for registering and using the program. The participants can contact the therapist *via* the web-based program at any time and all messages will be replied to within 1 week. Additional automatic reminders will be sent if a participant has not been active. If necessary, the therapists will call participants who have discontinued the program. They can track progress and read the answers to all the program’s written exercises and tasks.

**Table 2:** Summary of contents of the web-based program in use from June 2021 onward.

Module	Theme and aims	Examples of exercises
1. Module	**Introduction and bodily symptoms.** A brief introduction to the program including practical information. Information on central nervous system functioning.	“Ready for change” questionnaire, progressive relaxation^a^ ***Homework*:** Progressive relaxation exercise begins ^a^
2. Module	**Stress system.** The aim is to get information about the stress system and identify the factors and acts that increase or decrease wellbeing.	“Tug-of-war” metaphor, “Mindful breathing” exercise ***Homework*:** progressive relaxation exercise continues^a^, “Mindful breathing” exercise, taking action to increase wellbeing
3. Module	**Learning how our thoughts, emotions, bodily sensations, and behaviors influence each other.** The aim is to increase understanding of language as a double-edged sword and start defusing the content of thoughts and understand the effect of focusing attention.	“Leaves in a stream” exercise, mind mapping factors that influence on one’s wellbeing, “Activating event – thoughts and beliefs – consequences” exercise ***Homework*:** … “Activating event – thoughts and beliefs – consequences” exercise, Progressive relaxation exercise: short relaxation^a^
4. Module	**Automatic thoughts. The aim is to** identify automatic thoughts and assess destructive thought patterns and handle automatic thoughts. The aim is to increase understanding of language and continue defusing the content of thoughts.	*‘Cognitive distortions exercise* ***Homework*:** Continue with the “Activating event – thoughts and beliefs – consequences” exercise, Progressive relaxation exercise: short relaxation
5. Module	**Worrying and avoidance strategies.** The aim is to find alternative actions for unhelpful safety behavior such as experiential avoidance and worrying.	‘Warm donuts’ exercise, Chain analysis of core beliefs ***Homework*:** “Worry time” exercise, Progressive relaxation exercise: short relaxation^a^, “Mindful breathing” exercise
6. Module	**Emotions and the body.** The aim is to gain more understanding emotions and learn about observing and describing**e**motions, and emotion regulation.	“The observer” exercise, “The sky and the weather” metaphor ***Homework*:** “contacting the present moment” exercise, breathing exercise, additional relaxation exercise
7. Module	**Thoughts and emotions as a guide**. The aim is to increase defusion skills. Self-as-context is also discussed.	“Navigator” metaphor, “Leaves in a stream” exercise, cognitive defusion methods ***Homework*:** mindful walk, “Gentle hand” exercise
8. Module	**Self-perception and identity.** The aim is to increase self-perception and self-compassion by assessing literal rules and their consequences and interpersonal relationships.	“Gentle hand” exercise and reflection task on important relationships, “My 80^th^ birthday” exercise ***Homework*:** Mindful walk, “Gentle hand” exercise
9. Module	**Values and compassion**. The aim is to clarify values, and to practice using acceptance-based strategies in relation to inner experiences.	Reflection task on values, “Beach ball” metaphor, exercise on acceptance, exercises on self-compassion and compassion toward others ***Homework*:** exercise on acceptance, “Gentle hand” exercise
10. Module	**Summary.** The aim is to review important content from each module with a reflection on progress. An individual plan for continuing practicing is outlined.	Review of the progress worksheet, “My plan” worksheet

a Following the procedure presented in applied relaxation training (Öst, 1987).

The web-based program is provided by the Hospital District of Helsinki and Uusimaa, which already provided brief online-based treatments for various physical and mental health conditions with trained therapists. These online treatments are available for all of Finland. During the study, the program is also available to patients other than the current study participants *via* referral from their medical doctor, in which case the intervention program is paid for by the patients’ municipality. The cost of the study participants’ use of the program is covered by the study. The content of the pilot program was developed by researchers at the University of Jyväskylä involved in this study, using expertise offered by FIOH (details for the pilot version are shown in the Supplementary material). The structure of the program was further improved after feedback during the first 11 months after launching the pilot version of the program and the improved version was launched in May 2021.

### 2.7. Treatment as usual

TAU comprises all the routine care that an individual receives when they present their symptoms at the primary or student health service or occupational health services unit (corresponds to primary care-level treatment) or another unit that recommends the study to the participant. Both the intervention arm and TAU arm participants will receive usual care, but TAU will be enhanced by the study intervention in the intervention arm. In practice, TAU may vary among the study participants based on their individual needs, for example, treatments for comorbid somatic diseases or psychiatric disorders with which this study will not interfere.

In addition to TAU, all the participants will be given a self-help and educational leaflet based on scientific knowledge related to their condition. This leaflet includes a short description of the health conditions and an explanatory description of the biopsychosocial perpetuating mechanisms of PPS. The leaflet also outlines self-help principles for participants (Table 3).

**Table 3:** Description of educational leaflet for study participants.

Contents	PPS related to indoor air	Chronic fatigue
Specific contents	- Description of multi-organ symptom profile and three main categories of IA-related symptoms (i) complaint reactions due to poor subjective IA, (ii) disease or building-related illness that may be caused by IA factors and (iii) PPS with an unclear cause but with a possible relation to IA*) - Main principles of managing symptoms associated with IA - Factors related to build environment, psychosocial and personal factors associated with IA symptoms	- Description of core symptoms, multi-organ symptom profile, and differential diagnosis related to situational fatigue - Main principles of managing chronic fatigue and its consequences for an individual’s life (i) accepting the condition, (ii) understanding one’s role in symptom management, (iii) recognizing the predisposing and perpetuating factors of the symptoms, and iv) working on the cognitions and emotions that might increase the symptom burden)
Common contents	- Interaction of biopsychosocial factors that influence PPS - Automatic central nervous system reactions, “flight or fight” mechanism - Healthcare treatment for PPS based on individual assessment - Reactive psychological distress associated with PPS - Principles for self-help and symptom management	

* The focus of this study.

IA, indoor air; PPS, persistent physical symptoms.

### 2.8. Outcomes

The primary outcome of this study is health-related quality of life (HRQoL) measured by the 15D questionnaire (Sintonen, 2001; Sintonen, 2013). The 15D is a utility-based generic, standardized measure, comprising the following 15 dimensions that describe physical, mental, and social wellbeing: mobility, vision, hearing, breathing, sleeping, eating, speech, excretion, usual activities, mental function, discomfort and symptoms, depression, distress, vitality, and sexual activity. Each dimension is graded by the respondent on a scale ranging between 1 (no perceived problems at all) and 5 (severe problems). Thus, the 15D can be used to measure a vast number of health states. We will use the 15D data to derive 15D overall scores with values from 1 (full health) to 0 (being dead), as well as to obtain dimensional symptom profiles. We will also measure secondary outcomes such as condition-specific outcomes (fatigue or symptoms related to various environmental factors), daily functioning (e.g., occupational, social, and cognitive functioning), cognitive and emotional functioning and psychiatric symptoms, and treatment satisfaction and changes that are experienced as negative by the participants related to the intervention (Table 4). We will also collect demographics and background information. Table 4 shows the description of the measurements and chronology of the assessments. The set of outcomes follows the recommendations of the European expert network of clinicians and researchers on persistent somatic symptoms (EURONET-SOMA) designed to harmonize core outcome domains in clinical trials on PPS (Rief et al., 2017). A random sample of volunteer participants will be interviewed at the end of the trial considering the treatment to gather qualitative data about treatment usability and acceptance.

**Table 4:** Outcomes and their assessment time schedule.

	**Time of measurement**					
**Assessment method**	**BL**	**0 W**	**6 W**	**14 W**	**6**	**12**
**Primary outcome**						
Health-related quality of life 15D instrument (15D; Sintonen, 2001; Sintonen, 2013)	X	X	X	X	X	X
**Background variables**						
Demographics (age, gender, marital status, education)	X					X
Daily exercise, diet, smoking, alcohol (Audit-C; Bush et al., 1998)	X					X
Social support and loneliness	X					X
Health, diagnosed diseases, and medication	X					X
Work characteristics	X					X
Symptoms related to environmental factors	X			X	X	X
Resiliency (SOC-3) and Personality Inventory (PK5; Konstabel et al., 2012, 2017)	X					X
Sleep quality, sleeping patterns	X			X	X	X
Fatigue	X					X
**Symptoms**						
Generalized Anxiety Disorder 7 (GAD-7) (Spitzer et al., 2006)	X			X	X	X
Insomnia Severity Index (ISI; Morin and Barlow, 1993; Bastien et al., 2001; Järnefelt and Hublin, 2012)	X			X	X	X
Patient Health Questionnaire-15 (PHQ-15)	X			X	X	X
Patient Health Questionnaire (PHQ-9; Kroenke et al., 2001; Kaila et al., 2012)	X			X	X	X
**Cognitive and emotional functioning**						
Acceptance and Action Questionnaire II AAQ-7 (Bond et al., 2011)	X	X	X	X	X	X
Comprehensive assessment of Acceptance and Commitment Therapy CompACT (Francis et al., 2016)						
Cognitive Fusion Questionnaire (CFQ-7)	X	X	X	X	X	X
Whiteley index 7 (Pilowsky, 1967)	X			X		X
White Bear Suppression Inventory (WBSI; Wegner and Zanakos, 1994)	X	X		X	X	X
Five Facet Mindfulness Questionnaire (FFMQ; Baer et al., 2006, 2008)	X	X				X
Toronto alexithymia scale (TAS-20; Bagby et al., 1994a,b)	X					
Illness Perception Questionnaire (Broadbent et al., 2006)	X		X	X		X
**Occupational, study and psychosocial functioning**						
Self-assessed current work or study ability on a scale 0–10**, own prognosis of work ability 2 years from now (Tuomi et al., 1998)	X			X	X	X
Self-assessed stress and recovery on a scale of 1–10	X			X	X	X
Daily functioning in three sub-domains (work, social life, home) on a scale of 1–10**	X			X	X	X
**Treatment satisfaction, adverse effects, and usage**						
*Working Alliance Inventory (Horvath and Greenberg, 1989)		X	X	X		
Treatment satisfaction (Seligman, 1995)		X	X	X		
Interview of treatment acceptance						X
Engagement to intervention: total number of logins and time during web-based program, modules, and exercises completed						
Reasons for discontinuing						

BL, questionnaire before recruitment interview; 0 W, questionnaire after recruitment clinical interview; 6 W, questionnaire 6 weeks after beginning of eHealth intervention; 14 W, questionnaire 14 weeks after 0 W questionnaire; 6, questionnaire 6 months after 0 W questionnaire; 12, questionnaire 12 months after 0 W questionnaire.

* In the eHealth intervention arm, the participants fill in the Working Alliance Inventory (WAI) for4 weeks from the beginning of the web-based program and after the last module.

** Scales will be used as a base for assessing disability related to the symptoms in recruitment interview.

In addition, we will ask for the participants’ permission to use and combine the registered information of the health check-up data with the study data. The Finnish national health registers will be used to collect data on outpatient visits (AvoHILMO data), data on inpatient care (HILMO data), and data on occupational health service use, and to collect information on prescribed and reimbursed prescription medicine purchases, rights for special reimbursement for medicines, and information on sickness and disability benefits and rehabilitation with diagnoses. This information will be used to assess the effectiveness of the rehabilitation program in reducing the burden of healthcare services and the social security system.

### 2.9. Participant timeline

Figure 1 outlines the participant flow, data collection, and intervention timeline. All participant candidates fill in a questionnaire before the inclusion interview. If the interview reveals exclusion criteria, the participant candidate is excluded. The participants are recruited between August 2020 and June 2022. The final follow-up results are expected 12 months after the last recruited participant enters the study.

### 2.10. Randomization and blinding

If the inclusion criteria are met, the participants are allocated to the control group or treatment group by a pre-programmed, SPSS software-generated random allocation sequence modified from Arifin (2012). The allocation sequence will be carried out and concealed by a researcher who is not otherwise involved in the FIOH trial. The allocation ratio will be 1:1 so that the number of participants with either indoor air-related persistent symptomatology or with chronic fatigue will be balanced in both groups. After the clinical interview, the eligible participants will be assigned to the next study arm. Once the participants have been randomized, the SS or KK will contact them by telephone and email to inform them of their allocation. At the same time, the participants will receive the educational leaflet by email.

As this study compares an eHealth intervention with TAU, it is not possible to blind the study participants. However, the therapists provide support for all the patients referred to a web-based intervention program and are not explicitly informed if a patient is referred from the study. To wit, study participants and patients using the web-based program for other medical reasons are treated similarly independently of the referring unit. The data analysts will be blinded to the intervention arms.

### 2.11. Data collection, management, and analysis

All the questionnaires are web-based, and participants reply through a secure Internet connection. The participants’ confidentiality is protected by an encryption key to personal details, and in the final data, an ID number created for this study will be used to distinguish the participants. All the linked data will be collected and stored *via* the FIOH server. Any identifiable information collected will remain confidential. Only non-identifiable data will be used in the data analysis and the reporting.

### 2.12. Sample size calculation

The planned sample size is 200 participants. The power calculation was determined using Gpower 3.1 for two groups with four measurement points (after the recruitment and 14-week and 6- and 12-month follow-ups). The power analyses for the interaction effect between group and time suggested that 124 participants would be needed (62 + 62) to achieve a low-medium effect size (equal to a between-group effect size of 0.30 using Cohen’s d) with alpha = 0.05, power = 0.80. We assumed that ∼20% (one of five) of the recruited patients would not meet the inclusion criteria when interviewed at FIOH. We further assumed that the follow-up attrition would be ≤20%. These assumptions were based on the conservative estimate of our previous study of a similar patient population (Selinheimo et al., 2020). These calculations led us to estimate that a sample size of N = 100 participants randomized into the study arms would be appropriate.

The sample size was calculated to detect a minimally important change between the study arms in primary outcome measure 15D, ranging from 0 (dead) to 1 (full health). As a measure of clinical significance, we used a minimally significant change of 0.015 (Alanne et al., 2015).

### 2.13. Plan of statistical methods

The descriptive statistics (frequencies, means, and SD) of the baseline and follow-up data will be analyzed and reported. The level of significance will be set at *p* < 0.05 for all analyses.

Possible between-group differences at baseline will be analyzed using the ANOVA or *t*-test and Chi-square test in SPSS. The primary outcomes for change within the intervention groups and the differences between the changes in the intervention group and control group will be analyzed using Mplus and structural equation modeling with full-information maximum likelihood estimation. Structural equation modeling is equivalent to the repeated-measures ANOVA analysis. However, it accounts for missing values at random (MAR) and allows the inclusion of all the available data. We will also analyze categorical outcomes using the Chi-square test or Fisher’s exact test.

We will conduct primary analyses of all the participants who have been randomized to the study conditions. Thus, we will use intent-to-treat analyses in the Mplus analysis of all the randomized participants, regardless of whether or not they dropped out of the study. We will use a separate analysis for the participants who complete the whole intervention program. Item-level missing or error values due to coding are not expected due to the computerized forced protocol for the questionnaire. We will report the results for those who completed the pilot version between 9/2020–5/2021 and the improved version of the program together and separately. Our power calculations were based on the primary outcome without subgroup analyses and thus we consider secondary analyses exploratory in nature. Considering our secondary aim, exploratory analyses are based on the theoretical model of psychological flexibility.

The statistical analyses will use the latest version of IBM-SPSS for Windows (SPSS Illinois, Chicago, Illinois, United States) software and version 8.4 of Mplus (Muthen and Muthen, 2012).

### 2.14. Monitoring

This study will be monitored by the steering group every 6 months. The steering group will evaluate recruitment proceedings and data management and if necessary, propose changes to the protocol (amendments before submission of this protocol are reported in this manuscript). The approval of the ethics committee will be requested in any case of a change of study protocol and the ClinicalTrials.gov registry will be informed. All the authors will be given access to the cleaned data sets. Generally, no harms are reported during psychotherapeutic interventions (van Dessel et al., 2014) and no side effects or serious risks have been reported from participating in eHealth interventions based on case formulation and ACT programs. However, if any should occur during the treatment, the participants will be offered individual counseling from medical professionals (AV, TP), and will be referred for relevant treatment elsewhere if considered appropriate. To control the possibility of harm, our study participants will also remain in their usual care during the eHealth intervention to prevent any possible unwanted effects during the web-based program. Any adverse effects reported by the participants will be recorded and reported in the trial publications. As we expect no harm related to the intervention, no separate data monitoring group will be considered. Neither will further auditing procedures of the trial be considered.

## 3. Discussion

Persistent physical symptoms associated with indoor environments and chronic fatigue syndrome have serious consequences for individuals’ daily functioning and quality of life. Thus far, information on the intervention components that alleviate the symptoms and reduce the negative effects of the conditions remains scarce, despite the urgent need for effective and acceptable interventions for patients. We aim to study whether individual video-based case conceptualization and personal goal-setting and a web-based ACT intervention can improve the daily functioning of symptomatic individuals.

The design of the study has several strengths. First, in addition to the multiple questionnaire measures of functioning, symptomatology, and psychological processes that capture several individual factors of wellbeing, we will also use national registers to obtain information on the use of healthcare services and social benefits to complete patient-reported outcomes. Together, the measurements will enable the exploration of individual factors’ associations with the acceptability and improvement of global functioning following the intervention. Second, the study will provide novel information on the effectiveness of case formulation and shared intervention goal-setting and an ACT web-based intervention to improve wellbeing among two groups—patients with chronic fatigue and persistent physical symptoms associated with indoor environments—that currently have few effective treatment options available in TAU settings. To the best of the authors’ knowledge, individual case formulation based on shared decision-making (SDM), combined with a web-based intervention has not been studied before in these focus groups, although SDM is suggested to improve adherence to the treatment when conditions are prolonged (Joosten et al., 2008). Third, as it uses video meetings for individual case formulation and goal-setting for the intervention and web-based intervention, the study can reach a representative sample of participants from all over the country. If the intervention proves to be effective, structured case conceptualization should be made more widely available. Moreover, it will enable the identification of specific change processes to help the future planning of rehabilitation programs for alleviating the disabling effect of chronic fatigue and symptoms associated with the indoor environment.

The study also has limitations. Firstly, study inclusion and exclusion will be based on structured video-based clinical interviews and self-reported measures, and recruitment will involve no clinical examinations. The inclusion interview does, however, aim to assess the possible variability of the clinical examinations of the individuals before the study and to refer the individuals for further examinations in cases of suspected untreated medical conditions. However, the interviews might be long for especially individuals with symptom profile related to chronic fatigue which might influence the acceptability of the study protocol. Individual needs are taken into account when settling the interview schedule and if needed, the interviews and also study questionnaires are possible to complete in several parts to prevent the burden related to the recruitment procedure. Furthermore, as participation in the study is recommended to the participants by their physicians, this may cause selection bias among individual physicians and even among participants, as intervention studies, in general, include participants who are willing to receive psychological treatments (van Dessel et al., 2014). The study neither has no active control group that would enable us to evaluate treatment effectiveness in relation to other supplementary interventions in addition to TAU procedures. Finally, the web-based program, independent of the case formulation, is available for individuals with persistent physical symptoms regardless of the study, which may challenge the recruitment goals of this study.

However, despite these limitations, this study will provide novel information on the acceptance of individual case formulation and goal-setting in a web-based program and on the influence of a personalized eHealth program on persistent somatic symptoms.

## 4. Ethics, data protection, and dissemination

The Ethics Committee of the Hospital District of Helsinki and Uusimaa, Finland, has granted approval for this study (number HUS 915/2020). The confidentiality of the participants is protected by an encryption key to personal data. The key is stored separately. All data are treated and implemented according to national data security laws. The EU General Data Protection Regulation, Finnish data protection laws, and FIOH’s data protection guidelines will be strictly followed regarding storing and processing the data. FIOH is the data controller. The study will be conducted and reported in accordance with the CONSORT-EHEALTH (Eysenbach and Group, 2011) statement (CONSORT extension for web-based interventions) and SPIRIT (Chan et al., 2013) guidelines. The results will be published in peer-reviewed journals and presented at conferences. All results will be reported without any identifiable personal information. In this trial, all data are collected for scientific research purposes only, and participation in the study will not affect the participants’ healthcare. The data will not be used for other purposes that are incompatible with the research purpose. The participants have the right to know what data are related to them and the researchers will contact the data protection officer if they receive a request to ensure the correct procedure of action.

## Ethics statement

The studies involving human participants were reviewed and approved: The Ethics Committee of the Hospital District of Helsinki and Uusimaa, Finland, approved the trial study protocol on 30th April, 2020 (ID: HUS/915/2020). The research permission was granted in 25.6.2020 (ID: HUS 60/20/2020). The Ethics Committee of the Hospital District of Helsinki and Uusimaa approved the protocol amendments (latest 8.9.2021).

## Author contributions

SS finalized the research plan in collaboration with KK, AV, MS, RL, and TP. SS wrote the first version of the manuscript. KK, AV, SL, TP, MS, and RL helped in drafting the manuscript. TP is the principal investigator of the study. All authors provided feedback on the drafts and critically revised and approved the final manuscript.

## Funding

This study was funded by Social Insurance Institution of Finland (KELA) (ref. 75/331/2019) and Helsinki University Hospital (HUS) EVO (TYH2019315).

## Conflict of interest

AV works as a part-time medical consultant at OP Insurance Ltd., and has worked as a part-time medical consultant at The Social Insurance Institution of Finland (Kela, until 30 November 2018). AV has been a substitute member of the medical expert group of the Unemployment Security Appeal Board (TTLK, in accordance with the Unemployment Security Commission Act) until 31 December 2017. MS works as a part-time medical adviser to the Finnish Patient Insurance Centre (in accordance with the Patient Injuries Act).

The remaining authors declare that the research was conducted in the absence of any commercial or financial relationships that could be construed as a potential conflict of interest.

## Publisher’s note

All claims expressed in this article are solely those of the authors and do not necessarily represent those of their affiliated organizations, or those of the publisher, the editors and the reviewers. Any product that may be evaluated in this article, or claim that may be made by its manufacturer, is not guaranteed or endorsed by the publisher.
