# Supplementary material for: A randomized controlled trial protocol for persistent physical symptoms associated with indoor environment or chronic fatigue: Effectiveness of video-based functional case conceptualization and web-program for improving quality of life
Source: Front Psychol. 2023 Jan 6;13:923532. doi: 10.3389/fpsyg.2022.923532 (PMC9853541; doi:10.3389/fpsyg.2022.923532)
Supplement: Supplementary file 1 [file Data_Sheet_1.PDF]

Supplemental material for *A randomized controlled trial protocol for persistent physical symptoms associated with indoor environment or chronic fatigue: Effectiveness of video-based functional case conceptualization and web-program for improving quality of life*

Supplement 1. Contents of the pilot version of the web-programme

The pilot version of web-based program was offered after the meeting with the psychologist. It consisted of six manualized modules at two-week intervals. Participants were instructed to complete each module during the first week and to continue to integrate the content into their daily lives during the second weeks. The modules included psychoeducation and experiential exercises and training aimed at improving wellbeing and psychological flexibility in accordance with the contextual behavioural approach to wellbeing (Supplemental Table 1). All the modules included experiential exercises.

Supplemental table 1. Summary of contents of web-based program during pilot version (in use until May 2021)

Module	Theme and aims	Examples of exercises
Introduction	Brief introduction to program including practical information.	'Ready for changes?' questionnaire
1. Module	**Self-evaluation and mindfulness**. The aim is to identify the factors that increase or decrease wellbeing. Mindfulness exercises are introduced. Daily progressive relaxation practice begins.	Worksheets on exploring and naming symptoms and unwanted thoughts and feelings, worksheets on identifying ways to increase wellbeing, 'Mindful breathing' exercise<br>***Weekly homework***: taking action to increase wellbeing, daily mindfulness exercise or daily progressive relaxation exercise[a]
2. Module	**Values and commitment to valued action.** The aim is to clarify values and identify actions that serve personal values. The module also includes an exercise on defusion and self-compassion. Daily progressive relaxation exercises continue.	Values worksheets, 'My 80th birthday' exercise, 'Gentle hand' exercise and 'Leaves in a stream' exercise<br>***Weekly homework***: 'Leaves in a stream' exercise, daily valued action, progressive relaxation exercise[a]
3. Module	**Defusion and creative hopelessness**. The aim is to increase understanding of language and start defusing the content of thoughts. The problem of controlling inner experiences is introduced. Daily relaxation practice continues using a shorter exercise.	Psychoeducation on learning via language, worksheets on identifying one's personal rules and stories about oneself, 'Warm donuts' exercise, 'Tug-of-war' metaphor, mindfulness exercise<br>***Weekly homework***: Practising noticing, valued action, short relaxation exercise[a]
4. Module	**Defusion and mindfulness**. The aim is to increase defusion skills and incorporate mindfulness exercises into daily activities. Self-as-context is also discussed. Daily relaxation practice continues using a shorter exercise.	'Navigator' metaphor, 'Leaves in a stream' exercise, 'The observer' exercise, mindfulness exercises<br>***Weekly homework***: mindfulness exercises, daily valued action, short relaxation exercise[a]
5. Module	**Acceptance and compassion**. The aim is to practice using acceptance-based strategies in relation to inner experiences. The module includes exercises on self-compassion and compassion towards others. Daily relaxation practice is continued with cued relaxation incorporated into the exercise.	'Beach ball' metaphor, exercise on acceptance, 'Gentle hand' exercise, reflection task on important relationships<br>***Weekly homework***: valued action, short relaxation exercise with cued relaxation[a], additional relaxation exercise
6. Module	**Summary.** The aim is to review important content from each module with reflection on progress and additional exercises on key elements of the program. An individual plan for continuing practising is outlined.	Review of progress, 'Going East' metaphor, reflection on values work, 'Thoughts in my pocket' exercise, 'Fish under water' metaphor, 'The observer' exercise, 'My plan' worksheet

[a] Following the procedure presented in applied relaxation training (Öst, 1987)
